# Flavonoids: an overview

**DOI:** 10.1017/jns.2016.41

**Published:** 2016-12-29

**Authors:** A. N. Panche, A. D. Diwan, S. R. Chandra

**Affiliations:** 1 Department of Bio-Engineering, Birla Institute of Technology, Mesra, Ranchi, Jharkhand 835215, India; 2 MGM's Institute of Biosciences and Technology, Mahatma Gandhi Mission, N-6, CIDCO, Aurangabad-431003, India

**Keywords:** Flavonoids, Structure and composition, Biological activity, Research trends, Future research directions, Aβ, amyloid protein, AChE, acetylcholinesterase, AD, Alzheimer's disease, BACE-1, β active site cleavage enzyme-1, BChE, butyrylcholinsterase, COX, cyclo-oxygenase, NDM-1, New Delhi metallo-β-lactamase-1, XO, xanthine oxidase

## Abstract

Flavonoids, a group of natural substances with variable phenolic structures, are found in fruits, vegetables, grains, bark, roots, stems, flowers, tea and wine. These natural products are well known for their beneficial effects on health and efforts are being made to isolate the ingredients so called flavonoids. Flavonoids are now considered as an indispensable component in a variety of nutraceutical, pharmaceutical, medicinal and cosmetic applications. This is attributed to their anti-oxidative, anti-inflammatory, anti-mutagenic and anti-carcinogenic properties coupled with their capacity to modulate key cellular enzyme function. Research on flavonoids received an added impulse with the discovery of the low cardiovascular mortality rate and also prevention of CHD. Information on the working mechanisms of flavonoids is still not understood properly. However, it has widely been known for centuries that derivatives of plant origin possess a broad spectrum of biological activity. Current trends of research and development activities on flavonoids relate to isolation, identification, characterisation and functions of flavonoids and finally their applications on health benefits. Molecular docking and knowledge of bioinformatics are also being used to predict potential applications and manufacturing by industry. In the present review, attempts have been made to discuss the current trends of research and development on flavonoids, working mechanisms of flavonoids, flavonoid functions and applications, prediction of flavonoids as potential drugs in preventing chronic diseases and future research directions.

Flavonoids are an important class of natural products; particularly, they belong to a class of plant secondary metabolites having a polyphenolic structure, widely found in fruits, vegetables and certain beverages. They have miscellaneous favourable biochemical and antioxidant effects associated with various diseases such as cancer, Alzheimer's disease (AD), atherosclerosis, etc.^(^^1^^–^^3^^)^. Flavonoids are associated with a broad spectrum of health-promoting effects and are an indispensable component in a variety of nutraceutical, pharmaceutical, medicinal and cosmetic applications. This is because of their antioxidative, anti-inflammatory, anti-mutagenic and anti-carcinogenic properties coupled with their capacity to modulate key cellular enzyme functions. They are also known to be potent inhibitors for several enzymes, such as xanthine oxidase (XO), cyclo-oxygenase (COX), lipoxygenase and phosphoinositide 3-kinase^(^^4^^–^^6^^)^.

In nature, flavonoid compounds are products extracted from plants and they are found in several parts of the plant. Flavonoids are used by vegetables for their growth and defence against plaques^(^^7^^)^. They belong to a class of low-molecular-weight phenolic compounds that are widely distributed in the plant kingdom. They constitute one of the most characteristic classes of compounds in higher plants. Many flavonoids are easily recognised as flower pigments in most angiosperm families. However, their occurrence is not restricted to flowers but are found in all parts of plants^(^^8^^)^. Flavonoids are also abundantly found in foods and beverages of plant origin, such as fruits, vegetables, tea, cocoa and wine; hence they are termed as dietary flavonoids. Flavonoids have several subgroups, which include chalcones, flavones, flavonols and isoflavones. These subgroups have unique major sources. For example, onions and tea are major dietary sources of flavonols and flavones.

Flavonoids play a variety of biological activities in plants, animals and bacteria. In plants, flavonoids have long been known to be synthesised in particular sites and are responsible for the colour and aroma of flowers, and in fruits to attract pollinators and consequently fruit dispersion to help in seed and spore germination, and the growth and development of seedlings^(^^9^^)^. Flavonoids protect plants from different biotic and abiotic stresses and act as unique UV filters^(^^10^^)^, function as signal molecules, allopathic compounds, phytoalexins, detoxifying agents and antimicrobial defensive compounds. Flavonoids have roles against frost hardiness, drought resistance and may play a functional role in plant heat acclimatisation and freezing tolerance^(^^11^^)^. Jorgensen^(^^12^^)^ has mentioned that the early advances in floral genetics were primarily due to mutation techniques making an impact on flavonoid-derived flower colours, and demonstrated that functional gene silencing in plants was associated with flavonoid biosynthesis. Flavonoids have been ascribed positive effects on human and animal health and the current interest is for disease therapy and chemoprevention. Currently there are about 6000 flavonoids that contribute to the colourful pigments of fruits, herbs, vegetables and medicinal plants. Dixon & Pasinetti^(^^13^^)^ reviewed plant flavonoids and isoflavonoids in detail and discussed their applications to agriculture and neurosciences in human beings. Kumar & Pandey^(^^14^^)^ reviewed the protective roles of flavonoids against human diseases as well as their functions in plants. Recently Panche *et al*.^(^^15^^)^, while reviewing AD and current therapeutic methods, discussed in detail uses of flavonoids as plant secondary metabolites for the treatment of AD and the mechanisms involved. In the present review, attempts have been made to discuss the current trends of research and development on flavonoids, their applications as dietary and health benefits along with broad classification and future research directions.

## Classification

**Fig. 1.**
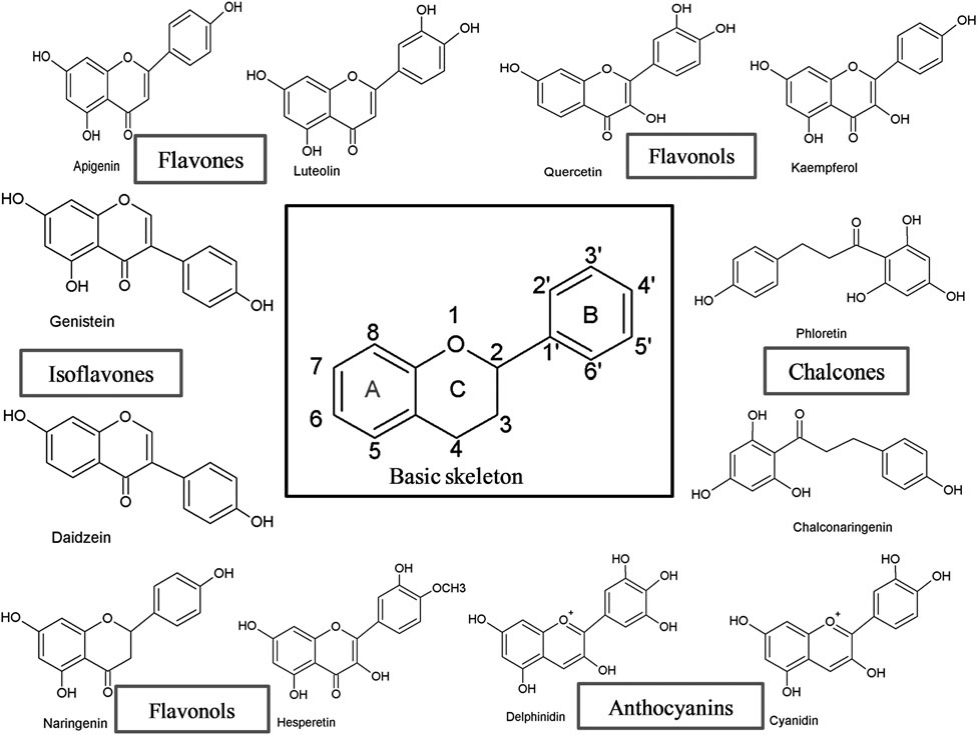
Basic skeleton structure of flavonoids and their classes.

Flavonoids can be subdivided into different subgroups depending on the carbon of the C ring on which the B ring is attached and the degree of unsaturation and oxidation of the C ring (Fig. 1). Flavonoids in which the B ring is linked in position 3 of the C ring are called isoflavones. Those in which the B ring is linked in position 4 are called neoflavonoids, while those in which the B ring is linked in position 2 can be further subdivided into several subgroups on the basis of the structural features of the C ring. These subgroups are: flavones, flavonols, flavanones, flavanonols, flavanols or catechins, anthocyanins and chalcones (Fig. 1).

### Flavones

**Fig. 2.**
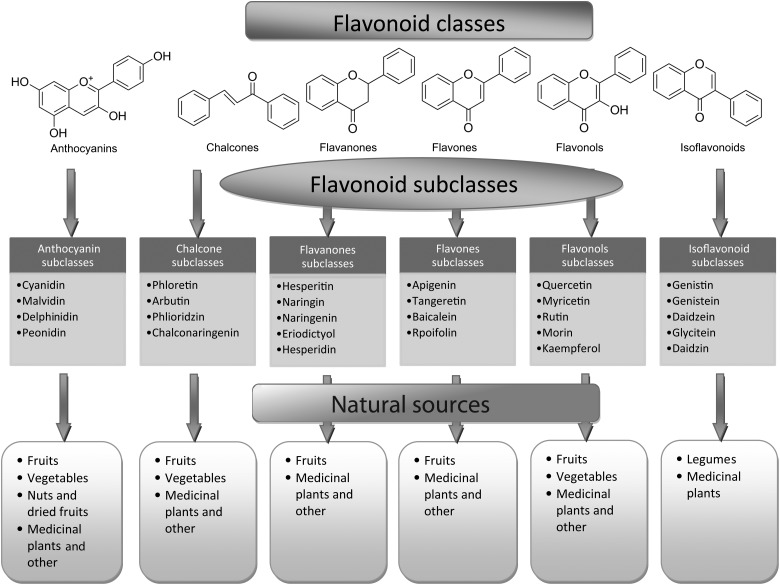
Flavonoid classes, subclasses and natural sources.

Flavones are one of the important subgroups of flavonoids. Flavones are widely present in leaves, flowers and fruits as glucosides. Celery, parsley, red peppers, chamomile, mint and ginkgo biloba are among the major sources of flavones. Luteolin, apigenin and tangeritin belong to this subclass of flavonoids (Fig. 2). The peels of citrus fruits are rich in the polymethoxylated flavones, tageretin, nobiletin and sinensetin^(^^16^^)^. They have a double bond between positions 2 and 3 and a ketone in position 4 of the C ring. Most flavones of vegetables and fruits have a hydroxyl group in position 5 of the A ring, while hydroxylation in other positions, for the most part in position 7 of the A ring or 3′ and 4′ of the B ring, may vary according to the taxonomic classification of the particular vegetable or fruit.

### Flavonols

Flavonols are flavonoids with a ketone group. They are building blocks of proanthocyanins. Flavonols occur abundantly in a variety of fruits and vegetables. The most studied flavonols are kaempferol, quercetin, myricetin and fisetin (Fig. 2). Onions, kale, lettuce, tomatoes, apples, grapes and berries are rich sources of flavonols. Apart from fruits and vegetables, tea and red wine are also sources of flavonols. Intake of flavonols is found to be associated with a wide range of health benefits which includes antioxidant potential and reduced risk of vascular disease.

Compared with flavones, flavonols have a hydroxyl group in position 3 of the C ring, which may also be glycosylated. Like flavones, flavonols are very diverse in methylation and hydroxylation patterns as well and, considering the different glycosylation patterns, they are perhaps the most common and largest subgroup of flavonoids in fruits and vegetables. For example, quercetin is present in many plant foods^(^^17^^)^.

### Flavanones

Flavanones are another important class which is generally present in all citrus fruits such as oranges, lemons and grapes. Hesperitin, naringenin and eriodictyol are examples of this class of flavonoids (Fig. 2). Flavonones are associated with a number of health benefits because of their free radical-scavenging properties. These compounds are responsible for the bitter taste of the juice and peel of citrus fruits. Citrus flavonoids exert interesting pharmacological effects as antioxidant, anti-inflammatory, blood lipid-lowering and cholesterol-lowering agents. Flavanones, also called dihydroflavones, have the C ring saturated; therefore, unlike flavones, the double bond between positions 2 and 3 is saturated and this is the only structural difference between the two subgroups of flavonoids. Over the past 15 years, the number of flavanones has significantly increased^(^^17^^)^.

### Isoflavonoids

Isoflavonoids are a large and very distinctive subgroup of flavonoids. Isoflavonoids enjoy only a limited distribution in the plant kingdom and are predominantly found in soyabeans and other leguminous plants. Some isoflavonoids have also been reported to be present in microbes^(^^18^^)^. They are also found to play an important role as precursors for the development of phytoalexins during plant microbe interactions^(^^19^^,^^20^^)^. Isoflavonoids exhibit tremendous potential to fight a number of diseases. Isoflavones such as genistein and daidzein are commonly regarded to be phyto-oestrogens because of their oestrogenic activity in certain animal models (Fig. 2). Szkudelska & Nogowski reviewed the effect of genistein inducing hormonal and metabolic changes, by virtue of which they can influence various disease pathways^(^^21^^)^.

### Neoflavonoids

Neoflavonoids are a class of polyphenolic compounds. While flavonoids have a 2-phenylchromen-4-one backbone, neoflavonoids have a 4-phenylchromen backbone with no hydroxyl group substitution at position 2. The first neoflavone isolated from natural sources in 1951 was calophyllolide from *Calophyllum inophyllum* seeds. It is also found in the bark and timber of the Sri Lankan endemic plant *Mesua thwaitesii*^(^^22^^–^^24^^)^.

### Flavanols, flavan-3-ols or catechins

Flavanonols, also called dihydroflavonols or catechins, are the 3-hydroxy derivatives of flavanones. They are a highly diversified and multisubstituted subgroup. Flavanols are also referred to flavan-3-ols as the hydroxyl group is always bound to position 3 of the C ring. Unlike many flavonoids, there is no double bond between positions 2 and 3. Flavanols are found abundantly in bananas, apples, blueberries, peaches and pears (Fig. 2).

### Anthocyanins

Anthocyanins are pigments responsible for colours in plants, flowers and fruits. Cyanidin, delphinidin, malvidin, pelargonidin and peonidin are the most commonly studied anthocyanins (Fig. 2). They occur predominantly in the outer cell layers of various fruits such as cranberries, black currants, red grapes, merlot grapes, raspberries, strawberries, blueberries, bilberries and blackberries. Stability coupled with health benefits of these compounds facilitate them to be used in the food industry in a variety of applications^(^^25^^)^. The colour of the anthocyanin depends on the pH and also by methylation or acylation at the hydroxyl groups on the A and B rings^(^^17^^)^.

### Chalcones

Chalcones are a subclass of flavonoids. They are characterised by the absence of ‘ring C’ of the basic flavonoid skeleton structure shown in Fig. 1. Hence, they can also be referred to as open-chain flavonoids. Major examples of chalcones include phloridzin, arbutin, phloretin and chalconaringenin. Chalcones occur in significant amounts in tomatoes, pears, strawberries, bearberries and certain wheat products. Chalcones and their derivatives have garnered considerable attention because of numerous nutritional and biological benefits. Table 1 describes the food sources of all dietary flavonoids discussed throughout the article for their bioactivity and research trends^(^^26^^–^^63^^)^. The intake of flavonoids through food sources could be the simplest and safest way to combat diseases as well as modulate activities.

**Table 1.** Flavonoids, their classes and rich dietary sources

Serial no.	Flavonoid	Class	Dietary sources	References
1	Quercetin	Flavonols	Vegetables, fruits and beverages, spices, soups, fruit juices	Hertog *et al*.^(^^26^^)^; Justesen & Knuthsen^(^^27^^)^; Stewart *et al*.^(^^28^^)^; Zheng & Wang^(^^29^^)^
2	Rutin	Flavonols	Green tea, grape seeds, red pepper, apple, citrus fruits, berries, peaches	Atanassova & Bagdassarian^(^^30^^)^; Gudrais^(^^31^^)^; Chang *et al*.^(^^32^^)^; Malagutti *et al*.^(^^33^^)^
3	Macluraxanthone	Xanthones	*Maclura tinctoria* (Hedge apple), Dyer's mulberry	Khan *et al*.^(^^34^^)^
4	Genistein	Isoflavone	Fats, oils, beef, red clover, soyabeans, psoralea, lupin, fava beans, kudzu, psoralea	Thompson *et al*.^(^^35^^)^; Umpress *et al*.^(^^36^^)^; Krenn *et al*.^(^^37^^)^; Coward *et al*.^(^^38^^)^; Kaufman *et al*.^(^^39^^)^
5	Scopoletin	Coumarin	Vinegar, dandelion coffee	Gálvez *et al*.^(^^40^^)^
6	Daidzein	Isoflavone	Soyabeans, tofu	Zhang *et al*.^(^^41^^)^
7	Taxifolin	Flavanonol	Vinegar	Cerezoa *et al*.^(^^42^^)^
8	Naringenin	Flavanone	Grapes	Felgines *et al*.^(^^43^^)^
9	Abyssinones	Flavanone	French bean seeds	Rathmell & Bendall^(^^44^^)^; Cruickshank *et al*.^(^^45^^)^
10	Rutin	Flavonol	Citrus fruits, apple, berries, peaches	Cruickshank *et al*.^(^^45^^)^; Chang *et al*.^(^^32^^)^
11	Eriodictyol	Flavanone	Lemons, rosehips	Hvattum^(^^46^^)^
12	Fisetin	Flavonol	Strawberries, apples, persimmons, onions, cucumbers	Sahu *et al*.^(^^47^^)^
13	Theaflavin	Catechins	Tea leaves, black tea, oolong tea	Leung *et al*.^(^^48^^)^
14	Peonidin	Anthocyanidin	Cranberries, blueberries, plums, grapes, cherries, sweet potatoes	Truong *et al*.^(^^49^^)^
15	Diosmetin	Flavone	Vetch	Andreeva *et al*.^(^^50^^)^
16	Tricin	Flavone	Rice bran	Cai *et al*.^(^^51^^)^
17	Biochanin	Isoflavone	Red clover, soya, alfalfa sprouts, peanuts, chickpeas (*Cicer arietinum*), other legumes	Medjakovic & Jungbauer^(^^52^^)^
18	Hesperidin	Flavanone	Bitter orange, petit grain, orange, orange juice, lemon, lime	National Agricultural Library^(^^53^^)^; Khan *et al*.^(^^34^^)^
19	Epicatechin	Flavan-3-ols	Milk, chocolate, commercial, reduced fat	Arts *et al*.^(^^54^^)^
20	Myricetin	Flavonols	Vegetables, fruits, nuts, berries, tea, red wine	Ross & Kasum^(^^55^^)^; Basli *et al*.^(^^56^^)^
21	Taxifolin	Flavanonol	Citrus fruits	Grayer & Veitch^(^^57^^)^; Kawaii *et al*.^(^^58^^)^
22	Kaempferol	Flavonols	Apples, grapes, tomatoes, green tea, potatoes, onions, broccoli, Brussels sprouts, squash, cucumbers, lettuce, green beans, peaches, blackberries, raspberries, spinach	Calderon-Montaño *et al*.^(^^59^^)^; Liu^(^^60^^)^; Kim & Choi^(^^61^^)^
23	Luteolin	Flavones	Celery, broccoli, green pepper, parsley, thyme, dandelion, perilla, chamomile tea, carrots, olive oil, peppermint, rosemary, navel oranges, oregano	Kayoko *et al.*^(^^62^^)^; López-Lázaro^(^^63^^)^
24	Apigenin	Flavones	Milk, chocolate, commercial, reduced fat	Hertog *et al*.^(^^26^^)^

## Current research and trends on flavonoids

### Anti-cholinesterase activity

Acetylcholinesterase (AChE) is a key enzyme in the central nervous system and inhibition of it leads to increases of neural acetylcholine levels which is one of the therapies for symptomatic relief of mild to moderate AD^(^^64^^)^. Hence the inhibition of cholinesterases is one of the central focus for drug development to combat AD. A number of flavonoids have been reported for their anti-cholinesterase activity. The *in vitro* inhibitory studies done on various flavonoids like quercetin, rutin, kaempferol 3-*O*-β-d-galactoside and macluraxanthone showed that quercetin and macluraxanthone possess a concentration-dependent inhibition ability against AChE and butyrylcholinsterase (BChE)^(^^34^^)^. Macluraxanthone was found to be the most potent and specific inhibitor of both the enzymes with 50 % inhibitory concentration (IC_50_) values of 8·47 and 29·8 µm, respectively. The enzyme kinetic studies revealed that quercetin inhibited both the enzymes in a competitive manner whereas macluraxanthone was non-competitive against AChE and competitive against BChE. To get insight of the intermolecular interactions, molecular docking studies of these two compounds were performed at active sites of both the enzymes. The docking studies showed that macluraxanthone binds much more tightly with both the enzymes than that of quercetin. Sheng *et al*.^(^^65^^)^, while designing, synthesising and performing the evaluation of flavonoid derivatives as potent AChE inhibitors, observed that most of the flavonoid derivatives have properties of inhibitory activities to AChE. The most potent inhibitor, isoflavone derivative 10d, inhibits AChE with an IC_50_ of 4 nm, showing a high BChE:AChE inhibition ratio (4575-fold), superior to donepezil (IC_50_ = 12 nm, 389-fold). Molecular docking studies were also performed to explore the detailed interaction with AChE.

### Anti-inflammatory activity

COX is an endogenous enzyme which catalyses the conversion of arachidonic acid into prostaglandins and thromboxanes^(^^66^^)^. The enzyme exists in two isoforms, COX-1 and COX-2. COX-1 is a constitutive enzyme and is responsible for the supply of prostaglandins which maintain the integrity of the gastric mucosa and provide adequate vascular homeostasis whereas COX-2 is an inducible enzyme and is expressed only after an inflammatory stimulus^(^^67^^)^. The function of COX-2 is to synthesise prostaglandins for the induction of inflammation and pain^(^^68^^)^. The studies done by using *in silico* methods on the binding modes of flavonoids with COX-2 explored that some flavonols and flavones containing a 2, 3-double bond may act as preferential inhibitors of COX-2^(^^69^^)^. These observations were found for the flavonol, flavone, and flavanone or isoflavone classes. This discovery led to the development of selective COX-2 inhibitors which are a class of compounds with good anti-inflammatory activity and reduced gastrointestinal side effects. The commercially available flavonoids like silbinin, galangin, scopoletin, hesperitin, genistein, daidzein, esculatin, taxifolin, naringenin and celecoxib were also evaluated for COX-inhibitory activity^(^^70^^)^. The selected flavonoids showed higher binding energy ranging between −8·77 to −6·24 kcal/mol (–36·69 to –26·11 kJ/mol) when compared with that of the standard (−8·30 kcal/mol; –34·73 kJ/mol) which led to the development of potent COX inhibitors for the treatment of inflammation. Madeswaran *et al*.^(^^70^^)^ evaluated the COX-inhibitory activity of flavonoids using *in silico* docking studies. In this perspective, they used flavonoids like farobin-A, gericudranin-B, glaziovianin-A, rutin and xanthotoxin. Their docking results showed that all the selected flavonoids contributed better aldose reductase inhibitory activity because of their structural parameters. Hence, further deeper studies could develop potent aldose reductase inhibitors for the treatment of diabetes. Madeswaran *et al*.^(^^71^^)^ also reported *in silico* docking studies of lipoxygenase-inhibitory activity of commercially available flavonoids. In this perspective, they selected flavonoids like aromadedrin, eriodictyol, fisetin, homoeriodictyol, pachypodol, rhamnetin, robinetin, tangeritin, theaflavin and azelastine for investigation. It was observed that all the selected flavonoids contributed to lipoxygenase-inhibitory activity because of their structural parameters and the whole analysis could lead to the further development of potent drugs for the treatment of inflammation. Wu *et al*.^(^^72^^)^ worked on antiplatelet effects and selective binding of COX with flavonoids and lignans by using the molecular docking method. The flavonoids considered were ginkgetin, Taiwan-homo-flavone A, Taiwan-homo-flavone B and Taiwan-homo-flavone C and eight known lignans justicidin B, justicidin C, justicidin D, chinensinaphthol methyl ether, procumphthalide A, procumbenoside A and ciliatosides A and B from medicinal herbal plants, *Cephalotaxus wilsoniana* and *Justicia* species, respectively. Out of these flavonoids ginkgetin, Taiwan-homo-flavone C, justicidin B and justicidin D were found to be effective for antiplatelet effects.

### Steroid-genesis modulators

Abyssinones and related flavonoids can be used as potential steroid-genesis modulators against three enzymes 3β-hydroxysteroid dehydrogenase (HSD), 17β-HSD and aromatase of the steroid-genesis pathway^(^^73^^)^. The virtual screening experiment indicated higher affinity for flavonones than their respective chalcones. The flavonones possess consistent binding affinity to all the three enzymes used and are better steroidogenesis modulators in hormone-dependent cancer.

### Xanthine oxidase modulators

XO catalyses the conversion of hypoxanthine to xanthine and subsequently xanthine to uric acid. The increase of uric acid level in blood serum, which is called hyperuricaemia, can lead to major complications such as gout and kidney stones^(^^74^^,^^75^^)^. Alnajjar^(^^76^^)^ worked on natural flavonoids towards the discovery of a potential XO inhibitor. Licoisoflavone-A extracted from the roots of *Glycyrrhiza glabra* (liquorice) showed the most potent activity in the inhibition of XO. Umamaheswari *et al*.^(^^77^^)^ evaluated XO-inhibitory activity of flavonoids using *in silico* docking studies. The flavonoids butein, fisetin, diosmetin, tricetin, genistein, tricin, vitexycarpin, herbacetin, biochanin, rhamnetin, isorhamnetin, robinetin, peonidin and okanin were studied and it was found that all flavonoids exerted inhibition activity. The presence of a benzopyran ring in their basic nucleus would have contributed to its XO-inhibitory activity. This molecular docking analysis may further lead to the development of potent XO inhibitors for the prevention and treatment of gout and related inflammatory ailments. New drugs for the inhibition of the enzyme aldose reductase are in development and efforts are being made for their preclinical and clinical evaluation.

A novel approach emphasising the significance of natural products as a prime solution to unanswered questions like the treatment of the ‘silent killer’ ‘polycystic kidney disease’ (PKD) has been investigated^(^^78^^)^. The key protein, namely cystic fibrosis transmembrane conductance regulator (which is responsible for PKD), and its mutated three-dimensional structure were subjected to molecular docking and *in silico* toxicity studies with flavonoids from vegetable sources. The outcome indicated the possible application of flavonoids from vegetable sources as potential and natural therapeutic agents to combat PKD.

Lin *et al*.^(^^79^^)^ carried out *in vitro* kinetic studies of different flavonoids as inhibitors with various xanthine concentrations. *In vitro* studies and kinetic measurements of different flavonoids and various concentrations of xanthine were carried out^(^^79^^)^. Four potent XO inhibitors were found in 95 % ethanolic (v/v) gnaphalium affine extract. Among them, the flavone eupatilin exhibited the strongest inhibitory effect on XO compared with allopurinol, a known synthetic XO inhibitor. Apigenin, luteolin and 5-hydroxy-6, 7, 3′, 4′-tetramethoxyflavone also contributed to the inhibitory effect of gnaphalium affine extract on XO activity. This study provides a rational basis for the traditional use of gnaphalium affine against gout. The study on *in vitro* XO-inhibitory activity of the aglycone hesperetin and its glycosylated forms (hesperidin and G-hesperidin) and their effects on the plasma lipid profile and the oxidative–antioxidative system has been carried out in rats^(^^80^^)^. The concentrations of the major conjugated metabolites in rat plasma after oral administration of these compounds were also determined. It has been reported that hesperetin was found to have a stronger XO-inhibitor activity than the glycosylate derivatives.

### Countering antibiotic resistance

β-Ketoacyl acyl carrier protein synthase III (KAS III), which initiates fatty acid synthesis in bacteria, is a key target enzyme to overcome the antibiotic resistance problem. Lee *et al*.^(^^81^^)^, while working on the known flavonoid inhibitors of β-KAS III against the methicillin-resistant bacteria *Staphylococcus aureus*, found that flavonoids such as naringenin (5,7,4′-trihydroxyflavanone) and eriodictyol (5,7,3′,4′-tetrahydroxyflavanone) are potent antimicrobial inhibitors of *Staphylococcus aureus* KAS III. Ganugapati *et al*.^(^^82^^)^ worked on *in silico* modelling and docking studies of a superbug enzyme, namely New Delhi metallo-β-lactamase-1 (NDM-1), which is an enzyme found in *Escherichia coli*. It has been reported that this enzyme belongs to a B1 subclass of metallo β-lactamases and is known to induce resistance to standard intravenous antibiotics. Similar studies were carried out on inhibition of NDM-1 in superbugs by flavonoids using the technique of *in silico* molecular docking^(^^83^^)^. At present, there are no effective antibiotics against the NDM-1-positive pathogen and therefore this study provides clues to investigate the molecular basis of extended antibiotic resistance of NDM-1 and then accelerate the search for new antibiotics against the NDM-1-positive strain in clinical studies. Quercetin and its analogue penta-*O*-ethylquercetin were found to be potential inhibitors of NDM-1.

### Disease-combating activity

Ganugapati *et al*.^(^^84^^)^ studied green tea flavonoids as insulin mimetics. Diabetes mellitus is a metabolism disorder where glucose, a principal source of energy, cannot enter the cells due to deficiency of insulin. The study suggested that epicatechin acts as an insulin receptor activator and reduces the harmful effects of diabetes. Lu & Chong^(^^85^^)^ carried out the computational work to predict the binding modes of flavonoid derivatives with the neuraminidase of the 2009 haemagglutinin 1 neuraminidase (H1N1) influenza virus. They employed molecular dynamics simulation techniques to optimise the 2009 H1N1 influenza neuraminidase X-ray crystal structure. All the twenty flavonoid derivatives were found to be satisfactory in binding and inhibiting the activity of the virus. These findings may help to develop a potential drug form of the flavonoid derivatives for the treatment of H1N1 influenza disease. Cardenas *et al*.^(^^86^^)^ showed through a study on mice that apigenin, a dietary flavonoid, exerts immune-regulatory activity. The study carried out on NF-κB luciferase transgenic mice showed effective modulation of NF-κB with no effect on the rate of cell death, a decrease in lipopolysaccharide-induced apoptosis in lungs, and infiltration of inflammation, leading to re-establishment of normal lung architecture. These effects indicate the immune-regulatory roles of flavonoids. Kim *et al*.^(^^87^^)^ reported that a flavonoid-rich diet is associated with a reduced risk of CVD. The study focused on individual as well as total flavonoid diet effects. Higher flavonoid intake was found to be associated with the improved CVD risk factors. Mulvihill *et al*.^(^^88^^)^ focused on the ability of citrus flavonoids to modulate lipid metabolism and other metabolic parameters related to the metabolic syndrome. This is one of the recent trends which have focused on citrus flavonoids as potential therapeutic agents for the treatment of metabolic dysregulation. The observational studies done by Hügel *et al*.^(^^89^^)^ indicated that dietary flavonoids are associated with a decreased risk of hypertension and CVD. A diet rich in all flavonoid classes through herbs and beverages improves vascular health leading to a reduced risk of diseases. It has been observed that the consumption of them is associated with improvement in endothelial function via vascular endothelial nitric oxide synthase and protein kinase B (Akt) activation. The effect of regular quercitin intake on blood pressure in overweight and obese patients with pre-hypertension and stage I hypertension was studied in seventy patients. Ambulatory blood pressure and office blood pressure were measured. It was observed that the blood pressure level was reduced in patients with hypertension^(^^90^^)^.

Recently it has been reported that an apple of the type pelingo is rich in food components that can markedly inhibit i*n vitro* tumorigenesis and the growth of human breast cancer cells^(^^91^^)^. It was observed that pelingo juice induced cell accumulation in the G2/M phase of the cell cycle, autophagy, inhibition of extracellular signal-regulated kinases 1/2 (ERK1/2) activity and an increase in lipidated microtubule-associated protein-1 light chain-3β (LC3B). Hence it could be used as a source of bioactive compounds with potential chemopreventive activity. Through the review of randomised controlled trials, it has been observed that intake of purified and extract forms of anthocyanins leads to significant improvement in LDL-cholesterol with no adverse effects^(^^92^^)^. An *in vivo* study model of rats was used to examine the effect of fenugreek seeds on renal pathology in alcoholics^(^^93^^)^. The different concentrations of seeds and their exerted effects were checked through transmission electron microscopy. The results showed reduction in cell deterioration and improvement in renal morphology and function. A tannin-rich extract obtained from the pinhão (*Araucaria angustifolia*) seed was found to inhibit α-amylase^(^^94^^)^. The same extract was also examined for inhibition of pancreatic lipase. An effective level of inhibition was observed for pancreatic lipase also. The extract also showed a significant reduction in TAG levels in mice. These results indicate that tannin can be used as a potential molecule for anti-obesity^(^^95^^)^. An extract of mixed polyphenolic compounds of grape seeds was found to comprise the ability to inhibit the aggregation and oligomerisation of β-amyloid *in vitro* and also improve the behavioural deficits in a mouse model of AD^(^^96^^)^. Paris *et al*.^(^^97^^)^ worked on flavonoids which lower Alzheimer's amyloid protein (Aβ) production via a nuclear factor κ-light-chain-enhancer of activated B cells (NF-κB)-dependent mechanism. It is well known that AD is due to the accumulation of Aβ peptides and the presence of neurofibrillary tangles in the brain^(^^98^^,^^99^^)^. Aβ is believed to play an important role in AD and it has been shown that certain flavonoids such as genistein, quercetin, taxifolin, kaemferol, luteolin, apigenin, daidzein, aminogeneistein, and α- and β-napthofalvone can affect Aβ production. Recently, it was suggested that the Aβ-lowering properties of flavonoids are mediated by a direct inhibition of β active site cleavage enzyme-1 (BACE-1) activity, the rate-limiting enzyme responsible for the production of Aβ peptides^(^^97^^)^. It has been reported that a strong correlation exists between the inhibition of NF-κB activation by flavonoids and their Aβ-lowering properties, suggesting that flavonoids inhibit Aβ production in whole cells via NF-κB-related mechanisms. As NF-κB has been shown to regulate BACE-1 expression, it has been concluded that NF-κB-lowering flavonoids inhibit BACE-1 transcription in human neuronal cells. Shimmyo *et al*.^(^^100^^)^, while working on structure–activity relationships in cell-free, cell-based and *in silico* modes revealed novel pharmacophore features of flavonoids. Their results contributed to the development of new BACE-1 inhibitors by certain natural flavonoids (myricetin, quercetin, kaempherol, morin, apigenin) for the treatment of AD. Swaminathan *et al*.^(^^101^^)^ worked on a series of natural and synthetic flavones and flavonols to explore their activity against radio ligand binding at human cloned muscarinic receptors. It has been mentioned that muscarinic acetylcholine receptor-active compounds have potential to treat AD^(^^102^^)^. Their findings indicated that there are several flavonoid compounds which possess competitive binding affinity, comparable with that of acetylcholine. Molecular modelling studies suggested that the compounds bind to the orthosteric site of the receptor, mainly through non-polar interactions. Further, it is mentioned that due to limitations in the docking and scoring functions used, no significant energy differences were observed for binding of the active compounds compared with the inactive compounds. These results give an indication of the potential of flavonoid compounds for the treatment of AD.

### Flavonoid mechanisms

Almost every group of flavonoids has a capacity to act as antioxidants. It has been reported that the flavones and catechins seem to be the most powerful flavonoids for protecting the body against reactive oxygen species. Body cells and tissues are continuously threatened by the damage caused by free radicals and reactive oxygen species, which are produced during normal oxygen metabolism or are induced by exogenous damage^(^^103^^,^^104^^)^. The mechanisms and the sequence of events by which free radicals interfere with cellular functions are not fully understood, but one of the most important events seems to be lipid peroxidation, which results in cellular membrane damage. This cellular damage causes a shift in the net charge of the cell, changing the osmotic pressure, leading to swelling and eventually cell death. Free radicals can attract various inflammatory mediators, contributing to a general inflammatory response and tissue damage. To protect themselves from reactive oxygen species, living organisms have developed several effective mechanisms^(^^105^^)^. The antioxidant defence mechanisms of the body include not only the enzymes such as superoxide dismutase, catalase and glutathione peroxidase, but also non-enzymic counterparts such as glutathione, ascorbic acid and α-tocopherol. The increased production of reactive oxygen species during injury results in consumption and depletion of the endogenous scavenging compounds. Flavonoids may have an additive effect to the endogenous scavenging compounds^(^^106^^)^. Codorniu-Hernández *et al*.^(^^107^^)^ carried out docking studies to understand flavonoid–protein interactions. The results indicated that hydrophilic amino acid residues demonstrate high-affinity interactions with flavonoid molecules, as was predicted by the theoretical affinity order. The docking modes among catechin molecules and four proteins (human serum albumin, transthyretin, elastase and renin) are also supporting this information. The theoretical affinity order among flavonoids and amino acid residues seems to have great applications in the theoretical predictions of flavonoid–protein interactions as a high-quality approach to understand the biological activity of flavonoids.

### Radical scavenging

Flavonoids can prevent injury caused by free radicals in various ways and one way is the direct scavenging of free radicals. Flavonoids are oxidised by radicals, resulting in a more stable, less-reactive radical. In other words, flavonoids stabilise the reactive oxygen species by reacting with the reactive compound of the radical. Because of the high reactivity of the hydroxyl group of the flavonoids, radicals are made inactive, as explained in the following equation as given by Korkina & Afanasev^(^^108^^)^:

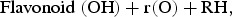

where R is a free radical and O is an oxygen free radical. Hanasaki *et al*.^(^^109^^)^ found that some of the flavonoids can directly scavenge superoxides, whereas other flavonoids can scavenge the highly reactive oxygen-derived radical called peroxynitrite. They found that flavonoids such as epicatechin and rutin are powerful radical scavengers and the scavenging ability of rutin may be due to its inhibitory activity on the enzyme XO. Kerry & Abbey^(^^110^^)^ reported that by scavenging radicals, flavonoids can inhibit LDL oxidation in *in vitro* studies. They further mentioned that this action protects the LDL particles and, theoretically, flavonoids may have preventive action against atherosclerosis.

### Xanthine oxidase inhibition

Sanhueza *et al*.^(^^111^^)^ worked on changes in the xanthine dehydrogenase:XO ratio in the rat kidney subjected to ischaemia–reperfusion stress and also studied the preventive effect of some flavonoids. They mentioned that the XO pathway is an important route in the oxidative injury to tissues, especially after ischaemia–reperfusion. Xanthine dehydrogenase is the form of the enzyme present under physiological conditions, but its configuration is changed to XO during ischaemic conditions. XO is a source of oxygen free radicals. In the reperfusion phase (reoxygenation), XO reacts with molecular oxygen, thereby releasing superoxide free radicals. Two flavonoids, quercetin and silibin, were found to inhibit XO activity, thereby resulting in decreased oxidative injury^(^^112^^,^^113^^)^. Cos *et al*.^(^^114^^)^ worked on structure–function relations in which the flavonoid luteolin (tetrahydroxyflavone) was reported to be the most potent inhibitor of XO.

### Anti-inflammation

Nijveldt *et al*.^(^^106^^)^ reported about how immobilisation of leucocytes in the blood vascular system can damage tissues through the release of oxidants and inflammators. They mentioned in their paper that the immobilisation and firm adhesion of leucocytes to the endothelial wall lead to the formation of oxygen-derived free radicals and also release of cytotoxic oxidants and inflammatory mediators. Under normal conditions, leucocytes move freely along the endothelial wall. However, during ischaemia and inflammation, various endothelium-derived mediators and complement factors may cause adhesion of the leucocytes to the endothelial wall, thereby immobilising them and stimulating degranulation of the neutrophil. As a result, oxidants and inflammatory mediators are released, resulting in injury to tissues. Friesenecker *et al*.^(^^115^^)^, while working on the oral administration of a purified micronised flavonoid fraction, found that the flavonoids suppresses leucocyte adhesion in ischaemia–reperfusion injury in hamsters. The decrease in the number of immobilised leucocytes by flavonoids may be related to the decrease in total serum complement and is a protective mechanism against inflammation-like conditions associated with reperfusion injury^(^^116^^)^. Some flavonoids have been shown to inhibit degranulation of neutrophils without affecting superoxide production^(^^117^^)^.

Compared with the research work done on the antioxidant capacities of flavonoids, there has been relatively little research on other possible mechanisms. One such mechanism by which flavonoids act is through interaction with various enzyme systems. Furthermore, some effects may be a result of a combination of radical scavenging and an interaction with enzyme functions^(^^107^^)^. Alcaraz & Ferrandiz^(^^118^^)^, while working on anti-inflammatory activity and the inhibition of arachidonic acid metabolism by flavonoids, reported that flavonoid inhibit the metabolism of arachidonic acid through the enzyme pathway. This feature gives flavonoids anti-inflammatory and anti-thrombogenic properties. The release of arachidonic acid is a starting point for a general inflammatory response and neutrophils containing lipoxygenase create chemotactic compounds from arachidonic acid.

### Combating neurodegenerative diseases

The recent studies on different plant metabolites have shown that flavonoids may perform a key role in enzyme and receptor systems of the brain, exerting significant effects on the central nervous system, like prevention of the neurodegeneration associated with AD and Parkinson's disease^(^^15^^,^^119^^)^. Flavonoids are capable of inhibiting enzymes, as there exist strong reports about inhibitory enzymes such as aldose reductase, XO, phosphodiesterase, Ca^2+^ ATPase, lipoxygenase and COX in preventive neurodegenerative diseases.

Considerable work has been carried out to search suitable and new flavonoids for therapeutic use in AD by using the technique of molecular docking. Hu *et al*.^(^^120^^)^ have designed a new series of flavonoids, evaluated them and discovered potent AChE inhibitors. Most of them showed more potent inhibitory activities against AChE than rivastigmine, an AD drug. Further, it was mentioned that the isoflavone skeleton would be a promising structural template for the development of novel AChE inhibitors. Khan^(^^34^^)^ has examined AChE- and BChE-inhibitory activities of four flavonoid derivatives –quercetin, rutin, kaempferol galactoside and macluraxanthone. Out of four flavonoids, macluraxanthone displayed a concentration-dependent inhibition of AChE and BChE. A number of flavonoids were studied to lower Alzheimer's Aβ production using molecular docking studies. It has been reported that there exists a strong correlation between flavonoids and inhibitions of NF-κB-related mechanisms. While doing work on the molecular docking of flavones as BACE-1 inhibitors, it has been found that the flavonoids potently inhibit BACE-1 activity through the interactions of flavonoids with the BACE-1 catalytic centre^(^^100^^)^.

## Functions and applications of flavonoids

**Fig. 3.**
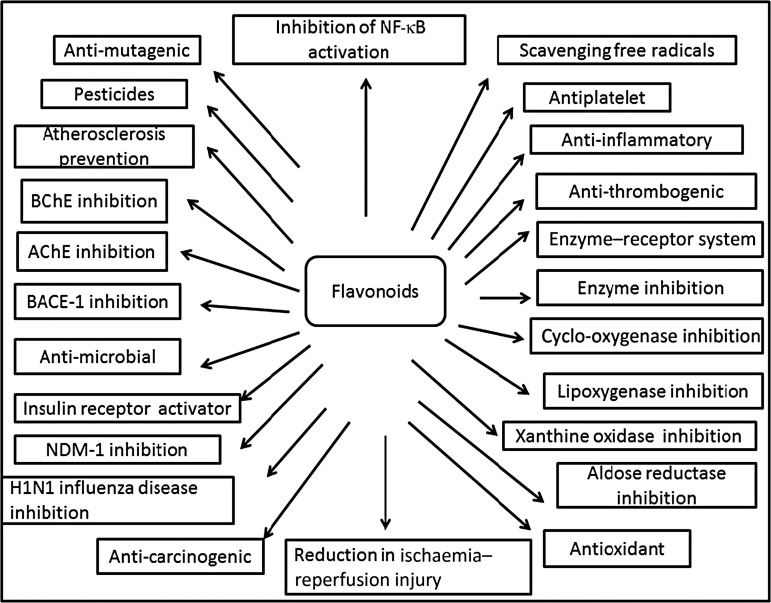
Cumulative representation of roles of flavonoids in various bioactivities, human health and agriculture. BChE, butyrylcholinesterase; AChE, acetylcholinesterase; BACE-1, β active site cleavage enzyme-1; NDM-1, New Delhi metallo-β-lactamase-1; H1N1, haemagglutinin 1 neuraminidase 1.

Plants produce a vast and diverse assortment of organic compounds, the great majority of which do not appear to participate directly in growth and development. These substances, traditionally referred to as secondary metabolites (flavonoids), often are differentially distributed among limited taxonomic groups within the plant kingdom^(^^121^^)^. The flavonoids are categorised in different classes as alkaloids, terpenoids and phenolics. Flavonoids carry out a number of protective functions in the human body (Fig. 3). Many flavonoids have evolved as bioactive compounds that interfere with nucleic acid or proteins and show antimicrobial or insecticidal and pharmacological properties. Flavonoids are therefore of interest in medicine as therapeutics and at the same instance in agriculture as pesticides^(^^122^^)^. *In vitro* technology has given new insight to explore the potency of plant cell tissue culture to produce the same valuable chemical compounds as those of the parent plant^(^^123^^)^. The advancement in plant tissue culture methods for flavonoid production has bloomed beyond expectations^(^^124^^)^. Plant tissue culture is an aseptic technique whereby proper manipulation of the nutrients, culture conditions, and phyto-hormone supply, one may be able to produce the desired quality and quantity of plants as well as metabolites. With the culture of differentiated cells it is possible to obtain production of the desired compounds in levels comparable with that of the plant. Flavonoids are associated with a broad spectrum of health-promoting effects. They are an indispensable component in a variety of nutraceutical, pharmaceutical, medicinal and cosmetic applications. This is attributed to their antioxidative^(^^125^^)^, anti-inflammatory^(^^126^^)^, anti-mutagenic^(^^127^^)^ and anti-carcinogenic^(^^128^^)^ properties coupled with their capacity to modulate key cellular enzyme functions^(^^129^^)^. Flavonoids act in plants as antioxidants, antimicrobials, photoreceptors, visual attractors, feeding repellents, and for light screening. Many studies have suggested that flavonoids exhibit biological activities, including anti-allergenic, antiviral, anti-inflammatory and vasodilating actions. However, most interest has been devoted to the antioxidant activity of flavonoids which is due to their ability to reduce free radical formation and to scavenge free radicals. The capacity of flavonoids to act as antioxidants *in vitro* has been the subject of several studies in the past years, and important structure–activity relationships of the antioxidant activity have been established^(^^125^^,^^130^^)^. Ren *et al*.^(^^130^^)^, in their paper on flavonoids and anticancer agents, gave the major molecular mechanisms of actions in different situations. In preventing carcinogens they mentioned that flavonoids exert their effects on cytochrome P450 to inhibit the activities of certain P450 isozymes which are responsible for the production of a number of procarcinogens. Another mechanism of action they reported is that flavonoids help in the production of metabolising enzymes such as gluthione-*S*-transferase, quinone reductase and uridine 5-diphospho-glucuronyl transferase by which carcinogens are detoxified and thus eliminated from the body. This would also help in preventing the chemotherapy effect of flavonoids against carcinogens.

A number of studies have been carried out on properties of antioxidant in relation to different flavonoids and these studies emphasised that the flavonoids can be used as potential drugs to prevent oxidative stresses^(^^131^^–^^136^^)^. Antioxidants are compounds that protect the cells against the oxidative effect of reactive oxygen species, and the impaired balance between these reactive oxygen species and antioxidants results in oxidative stress. The oxidative stress may lead to cellular damage which is related to various health ailments such as diabetes, cancer, CVD, neurodegenerative disorders and ageing. Oxidative stress can also damage many biological molecules and proteins and DNA molecules are significant targets of cellular injury. Antioxidants interfere with radical-producing systems and increase the function of endogenous antioxidants, protecting the cells from damage by these free radicals^(^^125^^)^. Pietta^(^^137^^)^ reviewed the current knowledge on structural aspects and *in vitro* antioxidant capacity of most common flavonoids as well as *in vitro* antioxidant activity and effects on endogenous antioxidants. Flavonoids have been found to be very effective in preventing lipid peroxidation and lipid peroxidation is responsible for various diseases such as atherosclerosis, diabetes, hepatotoxicity and inflammation, along with ageing^(^^138^^–^^140^^)^. Studies have indicated that quercetin helps to suppress lipid peroxidation^(^^141^^)^. In addition to quercetin, there are other flavonoids such as myricetin, quercetrin and rutin which help to inhibit the production of superoxide radicals^(^^103^^,^^104^^)^.

Flavonoids have also been recognised for their antimicrobial activity and many researchers have isolated and identified the structures of flavonoids having properties of antifungal, antiviral and antibacterial activity. Because of this property, many flavonoids are now being used extensively in the fields of nutrition, food safety and health. The antiviral effect of flavonoids has been shown by Wang *et al*.^(^^142^^)^, particularly in therapy for viral infection. Flavonoids such as quercetin, naringin, hesperetin and catechin possess a variable degree of antiviral activity. They affect the replication and infectivity of certain RNA and DNA viruses^(^^143^^)^. Quercetin and apigenin are among the most studied flavonoids which have been known to exhibit antibacterial activities^(^^144^^)^. Li & Xu^(^^145^^)^ have reported that quercetin extracted from lotus leaves may be a promising antibacterial agent for periodontitis.

Some flavonoids show hormone-like activities and they bear a resemblance to steroid hormones, particularly with oestrogen. Such flavonoids are present in fruits and vegetables, tea, red wine and cereals^(^^125^^)^. Hormone-like steroids are well known in protection against various chronic diseases, especially oestrogen, which has neuroprotective effects on the brain. A number of flavonoids such as genistein, daidzein and equol have been studied to assess their oestrogenic activity in clinical trials. The studies determined their potential for treatment of various chronic diseases such as cancer, cardiovascular disorders and osteoporosis^(^^146^^,^^147^^)^. From their studies it is found that the flavonoid genistein has the most promising effect in preventing postmenopausal bone loss in women. A number of flavonoids of dietary significance have been shown to impart beneficial impact on parameters associated with atherosclerosis, including lipoprotein oxidation, blood platelet aggregation and cardiovascular reactivity^(^^148^^,^^149^^)^. Comalada *et al*.^(^^150^^)^ reviewed the effects of flavonoids, particularly quercetin, on a variety of inflammatory processes and immune functions and it has been shown that certain flavonoids help in inhibiting the initial process of inflammation and improve the immune system. Anti-inflammatory activity using flavonoids and tannins from the leaves of the plant *Spilanthes paniculata* has been recently reported^(^^126^^)^. Anticancer effects of flavonoids such as tangeritin, 3-hydroxyflavone, 3′,4′-dihydroxyflavone, 2′,3′-dihydroxyflavone, fisetin, apigenin, luteolin daidzein and genistein have been carried out by a number of researchers^(^^151^^–^^154^^)^. Ren *et al*.^(^^130^^)^ and Huang *et al*.^(^^155^^)^, while working on natural phenolic compounds and their potential use for cancer prevention, reported that various flavonoids such as tannins, stilbenes, curcuminoids, coumarins, lignans, quinones and other flavonoids have chemopreventive properties and also contribute to induce apoptosis by arresting the cell cycle, regulating carcinogen metabolism and ontogenesis expression. While explaining the possible mechanism of flavonoids in cancer prevention they further mentioned that the flavonoids have complementary and overlapping mechanisms of action including antioxidant activity and scavenging free radicals, modulation of carcinogen metabolism, regulation of gene expression on oncogenes and tumour-suppressor genes in cell proliferation and differentiation, induction of cell cycle arrest and apoptosis, modulation of enzyme activities in detoxification, oxidation and reduction, anti-inflammatory properties and action on other possible targets. Flavonoids and their effect of protection of the central nervous system are concerned particularly with those related to neurodegenerative disease caused by the combined effect of oxidative stress, inflammation and transition metal accumulation; a good amount of information is available. Alzheimer's and related dementias are among some of the major disorders of neurodegeneration. Flavonoids, like flavonols, are associated with lower population rates of dementia^(^^156^^)^. Similarly, Hwang & Yen^(^^157^^)^ and Jager & Saaby^(^^119^^)^ suggested that citrus flavanones such as hesperidin, hesperetin and naringenin could traverse the blood–brain barrier and may play an effective role in the intervention for neurodegenerative diseases. The role of flavonoids in antidiabetic activity and anti-ageing has also been reported^(^^158^^–^^161^^)^.

## Future research and development programmes

Flavonoids have received much attention in the literature over the past 10 years and a variety of potential beneficial effects have been elucidated. However, a number of studies carried out involved *in vitro* and *in silico* studies. Therefore, further studies are needed so that the usefulness of flavonoids in the diet could be improved for better human health. The study of flavonoids is complex because of the heterogeneity of the different molecular structures and the scarcity of data on bioavailability. Furthermore, insufficient methods are available to measure oxidative damage *in vivo* and the measurement of objective end points remains difficult. There is a need to improve analytic techniques to allow the collection of more data on absorption and excretion. Data on the long-term consequences of chronic flavonoid ingestion are especially scarce. A number of reports have emphasised that molecular docking studies are required to identify the potential molecules of flavonoids for their usage in the treatment of various ailments in the human health system. The interactions of flavonoids with receptor molecules during the treatment of acute and chronic diseases are an important area of future research. More and more research is needed to discover new flavonoids from nature's bounty so that this will replace the use of synthetic medicines which are harmful to the body. In this context there is a need of research and development programmes involving *in vivo* studies which will give a hopeful and safe picture for the future. Currently, the intake of fruit, vegetables and beverages containing flavonoids is recommended, although it is too early to make recommendations on daily flavonoid intakes.
