# Flavonoids: an overview – CORRIGENDUM in Figure 1

**DOI:** 10.1017/jns.2024.73

**Published:** 2025-01-29

**Authors:** AN Panche, AD Diwan, SR Chandra

The author name Sheela Chandra was incorrectly noted as SR Chandra.

## Original text and correction:

**ORIGINAL Figure (Page 3, Figure 1)**

1. The structure of Hesperetin is not correct. Incorrect double bond between the 2,3 carbons and the alcohol group on the 3 carbon.
2. The example chalcones have identical structures. The structure of Chalconaringenin should have an alkene bond in it at the benzylic position to the phenol ring.
3. The example Flavonol of Quercetin should have an alcohol substituent at its 5 carbon according to the basic skeleton shown.
4. As the depicted ‘Anthocyanins’ are shown without glycosidic linkages, they may be more accurately described as ‘Anthocyanidins’.
5. The subclass of ‘Flavonols’ is listed twice; once in the top right, and once in the bottom left. The bottom left subclass should be listed instead as ‘Flavanones’ instead.

**CORRECTION**

Corrections made in figure (figure 1)

1. The structure of Hesperetin was corrected. Incorrect double bond between the 2,3 carbons was removed and the alcohol group on the 3 carbon replaced with O-CH_2_ bond.
2. The structure of Chalconaringenin edited with an alkene bond in it at the benzylic position to the phenol ring.
3. An alcohol substituent at 5 carbon of the Quercetin was added.
4. The ‘Anthocyanins’ without glycosidic linkages are described more accurately as ‘Anthocyanidins’.
5. The subclass of ‘Flavonols’ is listed twice; once in the top right, and once in the bottom left. The bottom left subclass was replaced as ‘Flavanones’.
